# Effect of dentine moisture conditions on interfacial adaptation of a calcium silicate-based sealer compared with an epoxy resin-based sealer: an SEM/EDX analysis

**DOI:** 10.2340/biid.v13.46517

**Published:** 2026-07-16

**Authors:** Maha M. Yahya, Baidaa Mohammed Zeidan, Njwan Fadhel Shehab

**Affiliations:** a Department of Conservative Dentistry, College of Dentistry, University of Mustansiriyah, Baghdad, Iraq; b Department of Conservative Dentistry, College of Dentistry, University of Mosul, Mosul, Iraq

**Keywords:** Adhesive interface, dentine moisture, EDX analysis, interfacial adaptation, sealer

## Abstract

**Introduction:**

To evaluate the effect of dentine moisture conditions on interfacial adaptation of a calcium silicate-based sealer (Bio-C Sealer) compared with that of an epoxy resin–based sealer (AH Plus Jet) at different root levels.

**Methods:**

Sixty extracted single-rooted teeth were prepared using ProTaper Universal system up to size F3 and randomly allocated to two groups (*n* = 30) according to the type of sealer. Each group was then further subdivided into three subgroups (*n* = 10 each) according to dentine moisture condition: dry (ethanol as final irrigant), normal moist (blotted dry), and wet (canals left flooded). Root sections (2 mm in thickness) were obtained at 3 mm (apical) and 8 mm (coronal). Interfacial adaptation was assessed using scanning electron microscopy (SEM) analysis, while elemental composition was analyzed utilizing energy-dispersive X-ray spectroscopy analysis (EDX). Data were analyzed using two-way analysis of variance and Tukey’s post hoc test (*p* ≤ 0.05).

**Results:**

Dentine moisture significantly affected interfacial adaptation. AH Plus Jet demonstrated superior adaptation under dry conditions, while Bio-C Sealer demonstrated optimal adaptation under moist conditions. EDX analysis revealed significantly higher levels of calcium and phosphorus in the Bio-C Sealer compared with the AH Plus group (*p* ≤ 0.019; *p* ≤ 0.036). Within the Bio-C Sealer group, calcium levels were significantly lower coronally (*p* = 0.016) than apically. Silicon and carbon were higher in AH Plus (*p* < 0.01), while oxygen was higher in Bio-C (*p* < 0.001).

**Conclusion:**

Dentine moisture conditions significantly influence the performance of sealer. AH Plus performs optimally under dry conditions, while Bio-C Sealer demonstrates superior performance in moist environments.

## Introduction

Based on the classification of endodontic materials, epoxy resin-based sealers, particularly AH Plus Jet (Dentsply Sirona; Ballaigues; Switzerland), have been considered as the gold standard due to their reliable clinical performance and favorable physicochemical properties [1]. Despite these advantages, this sealer exhibits intrinsic biological and interfacial limitations that warrant critical evaluation [2, 3].

From an interfacial perspective, the hydrophobic nature of the epoxy resin-based sealers justifies a well-controlled dry environment to accomplish optimal marginal adaptation [4, 5]. Residual dentine moisture may interfere with the polymerization, minimize penetration of sealers into the dentinal tubules, and promote interfacial gap formation [6]. Such limitations have led to the development of bioactive alternatives, especially calcium silicate-based sealers, which are formulated to function optimally in the presence of moisture. Calcium silicate-based (bioceramic) sealers interact with the dentinal tissues through hydration reactions that benefit from environmental moisture to produce calcium silicate hydrate and calcium hydroxide. These reaction products enhance the formation of apatite-like precipitates at the dentine–sealer interface, thus improving the biological seal and interfacial integrity [7].

Among calcium silicate-based sealers, Bio-C Sealer (Angelus; Londrina, PR, Brazil) has gained significant interest due to its premixed formulation and bioactive characteristics [8]. Previous studies have recorded its ability to penetrate into the dentinal tubules and promote mineralized interfacial layer formation, potentially enhancing its ability to seal the root canal system [9–12]. Despite these favorable properties, the performance of calcium silicate-based sealers is greatly dependent on the moisture condition of the root canal. Variations in dentine moisture, ranging from dry to moist or excessively wet, may affect hydration kinetics, ion release, and subsequent bio-mineralization [13, 14]. While some researchers have investigated the sealing ability and bonding strength of bio-ceramic sealers [14, 15] and their biocompatibility [16], limited data are available regarding the combined morphological and elemental characteristics of the dentine–sealer interface under different dentine moisture conditions.

Additionally, although the bioactivity of calcium silicate-based sealers has been widely investigated [14–17], limited evidence is available with regard to how controlled dentine moisture may affect their interfacial behavior in comparison with a conventional epoxy resin-based sealer. Given that clinical canal drying protocols vary and that the bio-ceramic materials differ fundamentally from resin-based sealers in their hydration interaction with dentine moisture, understanding these interactions is essential.

Thus, this study aimed to assess the influence of dentine moisture (dry, normal moist, and wet) on the interfacial adaptation and elemental composition of a calcium silicate-based sealer (Bio-C Sealer) in comparison to an epoxy resin-based sealer (AH Plus Jet) at the apical and coronal root canal levels, using combined scanning electron microscopy/energy-dispersive X-ray spectroscopy (SEM/EDX) analysis. The tested null hypotheses were:

1. Dentine moisture condition would not significantly influence the interfacial adaptation or elemental composition of the investigated sealers.
2. No significant differences would exist between the two root canal sealers in relation to interfacial adaptation and elemental composition, regardless of the dentine moisture condition or root canal region.

## Materials and methods

### Sample size determination

Sample size was calculated using G*Power software (version 3.1.9.7; Heinrich-Heine-Universität, Düsseldorf, Germany) based on a two-way analysis of variance (ANOVA) design. Assuming a medium effect size (*f* = 0.30), a statistical power of 0.80, and a significance level of *α* = 0.05. The minimum required sample size was 60 samples.

Sixty single-rooted human mandibular premolars with fully developed apices were collected from patients aged 18–35 years following extraction for orthodontic reasons. The teeth were cleaned of debris and stored in 0.1% thymol solution at 4°C for a maximum of 3 months to maintain dentine hydration [5].

### Root canal preparation

Teeth were sectioned at the level of the cemento-enamel junction using a low-speed diamond saw (Isomet 4000; Buehler, USA) under water cooling to obtain a standardized root lengths of 14 ± 1 mm. Working length was determined by inserting a #10 K-file until it became visible at the apical foramen, and then 1 mm was subtracted from the measured root length. All root canals were instrumented using the ProTaper Universal rotary system up to size F3 (30/.09) [5]. Irrigation protocol was performed by using 3 mL of 5% sodium hypochlorite (NaOCl) after each file with a 30-gauge side-vented needle, followed by 5 mL of 17% Ethylenediaminetetraacetic acid (EDTA) that was applied for 3 minutes to remove a smear layer, and then a final flush with distilled water was used [9].

### Dentinal moisture protocols

The manufacturer-reported classification and the chemical composition of the investigated sealers are detailed in Table 1.

**Table 1:** Classification and chemical composition of the investigated sealers.

Sealer	Manufacturer	Classification	Chemical composition
AH Plus Jet	Dentsply Sirona; Ballaigues; Switzerland	Epoxy resin-based root canal sealer	Paste A: Bisphenol-A epoxy resin, calcium tungstate, bispheno-F epoxy resin, silica, zirconium oxide, iron oxide pigments. Paste B: Dibenzyl diamine, calcium tungstate, tricyclodecane-diamine, aminoadamantane, silica, zirconium oxide silicone oil.
Bio-C Sealer	Angelus; Londrina; PR, Brazil	Premixed calcium silicate-based bioceramic root canal sealer	Calcium silicates, zirconium oxide, silicon dioxide, calcium oxide, iron oxide, calcium aluminate, dispersing agents, and thickening agents.

The specimens were randomly allocated into two main groups (*n* = 30) according to the type of sealer tested: **Group A** (AH Plus Jet) and **Group B** (Bio-C Sealer). Each group was then further divided into three subgroups (*n* = 10 each) based on the dentine moisture condition, as follows:

1. **A1 and B1 (Dry):** Canals were dried by using sterile paper points followed by irrigation with 95% ethanol for 10 seconds and then re-dried with paper points.
2. **A2 and B2 (Normal moist):** Canals were blot dried by using sterile paper points until no visible moisture was detected [10].
3. **A3 and B3 (Wet):** Canals were left flooded with distilled water to simulate excessive dentine moisture [6].

## Root canal obturation

Obturation of the root canals was done by using the single-cone technique. A master gutta-percha cone corresponding to the final preparation size was coated with the assigned sealer and then inserted to the full working length. Specimens were subsequently stored at 37°C and 100% relative humidity for 7 days to ensure complete setting of the sealers.

### Specimen sectioning and preparation

Specimens were embedded in epoxy resin molds and then sectioned horizontally by using a slow-speed diamond saw under water cooling. Two sections of 2 mm thickness were obtained at 3 mm (apical level) and 8 mm (coronal level) from the apex of the roots. Sections were polished using silicon carbide papers (600- , 800-, and 1200-grit), followed by 0.05 μm alumina suspension. Specimens were ultrasonically cleaned in distilled water for 3 minutes and then air dried.

### Scanning electron microscopy analysis

The specimens were mounted on aluminum stubs and sputter-coated with gold. The dentine–sealer interface was evaluated using a scanning electron microscope (Quanta 250 FEG; FEI, Netherlands) operating in backscattered electron mode at 15 kV. Interfacial adaptation was assessed qualitatively utilizing SEM micrographs by recording the presence of interfacial gaps and evaluating the continuity of the sealer–dentine interface. Images were obtained at a standardized magnification of ×1200. The assessment was descriptive in nature based on a comparative observation between the tested groups.

### Energy-dispersive X-ray spectroscopy analysis

Elemental detection was conducted using the EDX system (Oxford Instruments, UK) attached to the SEM [18]. Three standardized regions were analyzed: the sealer–dentine interface, dentine side, and sealer side.

Weight (Wt%) and atomic (At%) percentages of the following elements were recorded: carbon (C), oxygen (O), silicon (Si), phosphorus (P), and calcium (Ca).

### Statistical analysis

Data were presented as mean ± standard deviation (SD). Statistical analysis was carried out by using IBM SPSS Statistics version 28.0 (IBM Corp., Armonk, NY, USA). The effects of sealer type and dentine moisture conditions were assessed utilizing two-way ANOVA followed by Tukey’s post hoc test for multiple comparisons when required. The level of significance was established at *p* < 0.05.

## Results

### Interfacial adaptation (SEM analysis)

**Figure 1:**
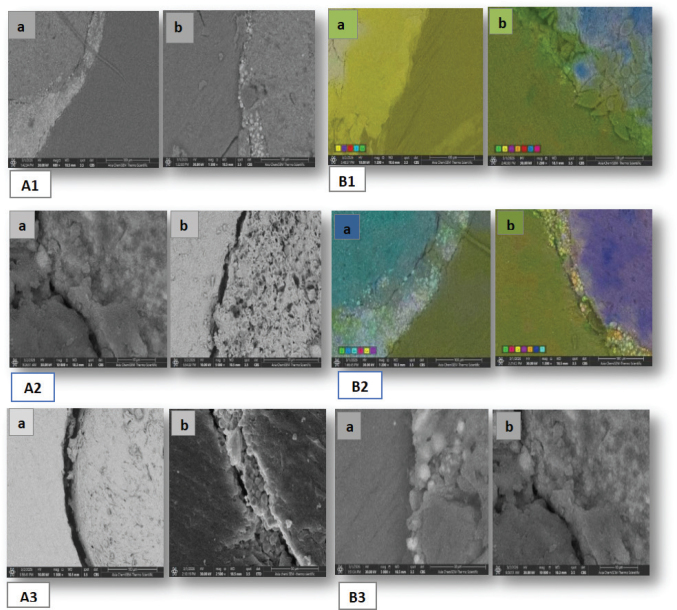
Representative SEM micrographs highlighting the sealer–dentine interface under three different dentine moisture conditions. A1–A3, respectively, show AH Plus under dry, normal moist, and wet conditions; B1–B3 represent Bio-C Sealer under the dry, moist, and wet conditions. Images marked (a) indicate the apical level and images marked and (b) indicate the coronal level. Dry-condition AH Plus specimens revealed fewer interfacial discontinuities, while the wet condition specimens recorded more visible gaps. Bio-C Sealer revealed the most favorable interfacial adaptation with the normal moist conditions. All micrographs were recorded at ×1200 magnification. SEM: scanning electron microscopy.

**Figure 2:**
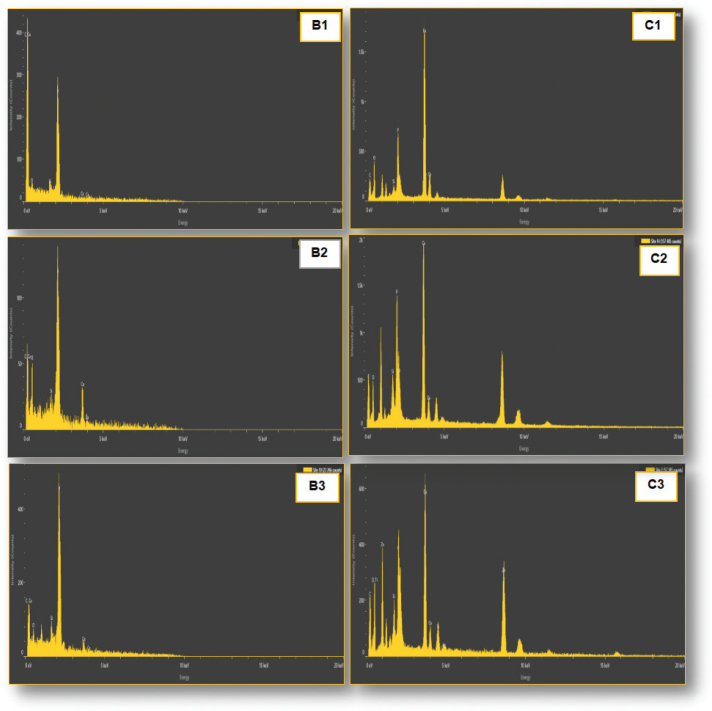
Representative EDX spectra of AH Plus under diverse moisture conditions: (A1) dry, (A2) normal moisture, and (A3) wet at the apical level, along with corresponding spectra at the coronal level (C1–C3), demonstrating the elemental composition across conditions. EDX: energy-dispersive X-ray spectroscopy analysis.

**Figure 3:**
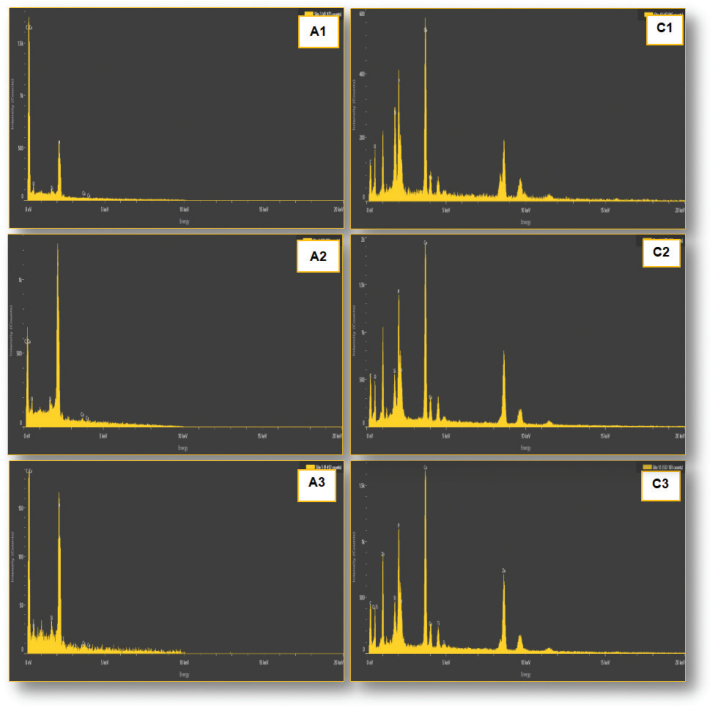
Representative EDX spectra of Bio-C Sealer under diverse moisture conditions: (B1) dry, (B2) normal moisture, and (B3) wet at the apical level, along with corresponding spectra at the coronal level (C1–C3), demonstrating the elemental composition across conditions. EDX: energy-dispersive X-ray spectroscopy analysis.

In Figures 1–3, SEM analysis demonstrated variations in interfacial adaptation among all tested groups. The AH Plus Jet sealer showed a more continuous sealer–dentine interface with fewer visible gaps under dry conditions (A1), while more defects (disruptions) were recorded under wet conditions (A3).

Bio-C Sealer recorded the most favorable interfacial integrity under normal moist conditions (B2), showing intimate contact with the dentine walls and fewer detected defects (interfacial gaps) when compared with the dry and wet conditions.

Dentine moisture significantly affected the interfacial integrity at both the apical and coronal segments. The AH Plus Jet Sealer demonstrated superior adaptation with minimal interfacial gaps under dry conditions (A1), while wet conditions (A3) exhibited the least favorable adaptation with increased gap formation, and the moist condition (A2) recorded intermediate performance.

Figure 1 demonstrated that Bio-C Sealer recorded optimal interfacial adaptation with intimate sealer–dentine contact and reduced interfacial defects (discontinuities) under moist conditions (B2). Under dry (B1) and wet (B3) conditions, an inferior interfacial adaptation was observed although wet conditions showed a slight deterioration irrespective of the root level.

### Elemental analysis

The quantitative elemental data are presented in Tables 2–4. For both sealers, EDX spectra showed distinct elemental profiles. Bio-C Sealer recorded higher calcium and phosphorus signals, while AH Plus Jet showed predominant silicon and carbon peaks (Figures 2 and 3). The results of Tukey’s post hoc tests are presented in Table 4.

**Table 2:** Mean ± standard deviation (SD) of elemental composition (Wt% and At%) at the apical level for all subgroups.

Elements	A1 Dry		A2 Moist		A3 Wet		B1 Dry		B2 Moist		B3 Wet	
	Wt% ± SD	At% ± SD	Wt% ± SD	At% ± SD	Wt% ± SD	At% ± SD	Wt% ± SD	At% ± SD	Wt% ± SD	At% ± SD	Wt% ± SD	At% ± SD
**C**	85.5 ± 0.9	90.60 ± 0.6	60.2 ± 0.2	73.2 ± 0.2	72.5 ± 0.5	80.1 ± 1.1	85.9 ± 0.4	89 ± 1.0	17.5 ± 0.5	29.7 ± 1.5	29.2 ± 0.2	46.2 ± 0.2
**O**	8.7 ± 0.1	6.90 ± 1.0	19.9 ± 0.1	18.2 ± 0.2	21.2 ± 0.2	17.6 ± 0.6	14.1 ± 2.4	11 ± 0.9	35.9 ± 0.9	45.8 ± 1.8	27.7 ± 1.5	32.9 ± 2.5
**Si**	4.60 ± 0.4	2.10 ± 0.1	9.2 ± 0.2	4.8 ± 0.25	1.5 ± 0.5	0.7 ± 0.05	-	-	3 ± 0.25	2.2 ± 0.2	1.7 ± 0.8	1.2 ± 0.2
**P**	Not detected	-	-	-	-	-	-	-	-	-	-	-
**Ca**	1.30 ± 0.05	0.40 ± 0.5	10.6 ± 0.4	3.9 ± 0.1	4.8 ± 0.2	1.6 ± 0.2	-	-	43.6 ± 0.65	22.2 ± 0.25	41.4 ± 0.4	19.7 ± 1.2

A1 = AH Plus (dry condition), A2 = AH Plus (normal moist condition), A3 = AH Plus (wet condition).

B1 = Bio-C Sealer (dry condition), B2 = Bio-C Sealer (normal moist condition), B3 = Bio-C Sealer (wet condition).

**Table 3:** Independent samples *t*-test comparing elemental composition between AH Plus and Bio-C Sealer at apical and coronal levels.

Element	Subgroups	Apical segment				Coronal segment			
		Weight %		Atomic %		Weight %		Atomic %	
		Mean diff	*P*	Mean diff	*P*	Mean diff	*P*	Mean diff	*P*
C	A1vsB1	-0.4	0.628	1.6	0.076	6.4	0.001*	10.1	0.000*
	A2vsB2	42.7	0.00*	43.5	0.00*	6.8	0.111	10.5	0.004*
	A3vsB3	43.3	0.00*	33.9	0.00*	1.6	0.012*	3.2	0.062
O	A1vsB1	-5.4	0.00*	-4.1	0.006*	-7.1	0.001*	-10.6	0.000*
	A2vsB2	-16	0.01*	-27.6	0.00*	-4.9	0.00*	-8.1	0.000*
	A3vsB3	-6.5	0.00*	-15.3	0.00*	-4.6	0.00*	-5.8	0.000*
Si	A1vsB1	4.6	0.00*	2.1	0.006*	6.5	0.00*	4.7	0.001*
	A2vsB2	6.2	0.00*	2.6	0.00*	4.2	0.276	1.6	0.014*
	A3vsB3	-0.2	0.708	-0.5	0.014*	0.6	0.37	0.5	0.30
P	A1vsB1	-	-	-	-	-0.8	0.217	-0.7	0.232
	A2vsB2	-	-	-	-	4.6	0.022	3.1	0.106
	A3vsB3	-	-	-	-	1.1	0.152	0.8	0.012*
Ca	A1vsB1	1.3	0.00*	0.4	0.005*	-6.2	0.00*	-4.7	0.005*
	A2vsB2	-33	0.00*	-18.3	0.00*	13.2	0.00*	6.4	0.000*
	A3vsB3	-36.6	0.00*	-18.1	0.00*	4.8	0.001*	2.6	0.004*

* Significant difference (*p* ≤ 0.05). **A1**: AH Plus in dry condition. **A2**: AH Plus in normal moist. **A3**: AH Plus in wet condition. **B1**: Bio-C Sealer in dry condition. **B2**: Bio-C Sealer in normal moist. **B3**: Bio-C Sealer in wet condition.

**Table 4:** Tukey’s post hoc test for multiple comparisons of elemental composition among different dentine moisture conditions.

Element	Apically					Coronally				
	Subgroups	Weight %		Atomic %		Subgroups	Weight %		Atomic %	
		Mean diff	*P*	Mean diff	*P*		Mean diff	*P*	Mean diff	*P*
C	A1vsA2	-3.8	0.006*	-7.7	0.337	B1vs B2	-3.4	0.00*	-7.3	0.836
	A1vsA3	-4.1	0.00*	-8.2	0.003*	B1vsB3	-8.9	0.623	-15.1	0.00*
	A2vsB3	-0.3	1.0	-0.5	0.264	B2vs B3	-5.5	0.029*	-7.8	0.044*
O	A1vsA2	5.6	0.036*	6.5	0.005*	B1vs B2	7.8	0.00*	9	0.01
	A1vsA3	6.1	0.00*	7.1	0.003*	B1vsB3	8.6	0.00*	11.9	0.00*
	A2vsB3	0.5	1.0	0.6	0.264	B2vs B3	0.8	0.065	2.9	0.044*
Si	A1vsA2	4.2	0.903	3	0.00*	B1vs B2	0.3	0.00*	0.3	0.002*
	A1vsA3	4.4	0.870	3.1	0.00*	B1vsB3	-1.5	0.00*	-1.1	0.00*
	A2vsB3	0.2	0.82	0.1	0.066	B2vs B3	-1.8	0.507	-1.4	0.005*
P	A1vsA2	0.3	0.00*	0.1	0.00*	B1vs B2	3.1	0.004*	2.1	0.120
	A1vsA3	0	0.00*	-0.1	0.00*	B1vsB3	1.9	0.00*	1.4	0.00*
	A2vsB3	-0.3	0.943	-0.2	0.174	B2vs B3	-1.2	0.011*	-0.7	0.028*
Ca	A1vsA2	4.5	0.511	2	0.00*	B1vs B2	1.4	0.00*	0.2	0.049
	A1vsA3	3.8	0.87	1.7	0.00*	B1vsB3	14.8	0.00*	7	0.00*
	A2vsB3	-0.7	0.997	-0.3	0.569	B2vs B3	13.4	0.01*	7.8	0.168

* Significant difference (*p* ≤ 0.05). **A1**: AH Plus in dry condition. **A2**: AH Plus in normal moist. **A3**: AH Plus in wet condition. **B1**: Bio-C Sealer in dry condition. **B2**: Bio-C Sealer in normal moist. **B3**: Bio-C Sealer in wet condition.

#### Carbon (C)

The AH Plus Sealer recorded a significantly higher carbon content when compared with Bio-C Sealer under all moisture conditions. At the apical level, carbon (wt%) in AH Plus varied from 85.5 ± 0.9 (A1) to 72.5 ± 0.5 (A3), while Bio-C Sealer showed lower values, in particular under normal moist conditions, 17.5 ± 0.5 (B2) and 29.2 ± 0.2 (B3). The statistical analysis indicated significant differences between respective subgroups, primarily A2 compared to B2 and A3 compared to B3 (*p* < 0.001), as recorded in Table 3.

#### Oxygen (O)

In Bio-C Sealer, the oxygen (O) levels were significantly higher compared with AH Plus. Bio-C Sealer recorded values of 35.9 ± 0.9 (B2) and 27.7 ± 1.5 (B3), while the AH Plus Sealer values ranging from 8.7 ± 0.1 (A1) to 21.2 ± 0.2 (A3). The differences were statistically significant for all study groups (*p* < 0.001) as shown in Table 3.

#### Silicon (Si)

AH Plus recorded a significantly higher silicon content. At the apical level, recorded values were 9.2 ± 0.2 (A2) and 4.60 ± 0.4 (A1), while lower values were recorded for Bio-C Sealer: 3.0 ± 0.25 (B2) and 1.7 ± 0.8 (B3). Statistically significant differences were demonstrated for most tested subgroups (*p* < 0.01) (Table 3).

#### Calcium (Ca)

Bio-C Sealer demonstrated significantly higher levels of calcium when compared with that of AH Plus Jet. At the apical level: Bio-C Sealer recorded values of 43.6 ± 0.65 (B2) and 41.4 ± 0.4 (B3), while the AH Plus Jet recorded values of 1.30 ± 0.05 (A1) to 10.6 ± 0.4 (A2). The differences were highly significant (*p* < 0.001) (Table 3).

#### Phosphorus (P)

Phosphorus was observed mainly in Bio-C Sealer, with higher values recorded under moist conditions. Statistically significant differences were demonstrated between tested groups, especially at the coronal level (*p* < 0.05) (Table 3).

### Impact of root level

Elemental distribution revealed variation between the evaluated apical and coronal levels. In Bio-C Sealer, the levels of calcium were significantly higher at the apical segment when compared with the coronal one (*p* = 0.016). On the other hand, no consistent regional differences in elemental composition were detected with AH Plus Jet.

## Discussion

This current investigation assessed the impact of dentine moisture on the interfacial integrity of a calcium silicate-based sealer compared with that of an epoxy resin-based sealer using SEM and EDX. Both hypotheses were rejected, highlighting that the type of sealer and dentine moisture condition significantly affected the outcomes. The results revealed that dentine moisture is a crucial factor affecting the interfacial performance of sealers, reflecting their various setting mechanisms and physicochemical properties [1, 18].

The single cone obturation technique was applied in this study to standardize the obturation procedure for all tested groups and to limit potential interfering factors associated with the compaction forces in addition to operator-related variables. Although clinically, AH Plus Jet is mainly used with compaction-based obturation techniques, using the exact obturation procedure in all study groups ensured a more reliable comparison of the impact of dentine moisture conditions on the interfacial integrity. Thus, any recorded variations could be more confidently related to the tested variables rather than the obturation technique.

In addition to the intrinsic properties of sealers, some methodological factors may have an impact on the observed findings. The obturation technique could affect the distribution of the root canal sealer and interface formation, especially when different materials exhibit distinct flow and setting behaviors.

The thickness of sealer may affect the setting mechanisms, interfacial integrity and dimensional stability, especially in materials that depend on hydration reactions. The storage conditions should also be considered, as temperature and humidity may affect the physicochemical behavior and the hydration process of calcium silicate-based root canal sealers [19]. These factors may partially explain the variations recorded between the tested sealers under different dentine moisture conditions.

Enhanced interfacial adaptation of AH Plus Jet root canal sealer under dry conditions can be ascribed to its hydrophobic epoxy resin matrix and moisture-sensitive polymerization reaction [1, 2]. Epoxy resin-based sealers undergo an epoxide-amine reaction that is water independent; however, the presence of residual moisture may interfere with polymer chain formation, reduce sealer penetration into the dentinal tubules, and may compromise interfacial integrity [19]. These findings are consistent with previous studies that reported improved bonding and marginal adaptation of AH Plus Jet in adequately dried canals [4, 5]. Conversely, increased moisture has been associated with compromised adhesion and greater interfacial gap formation [20, 21].

On the other hand, Bio-C Sealer revealed optimal interfacial adaptation under moist conditions, which is consistent with its hydration-dependent setting reaction [22, 23]. Calcium silicate-based sealers require moisture to initiate hydration reactions, leading to the formation of calcium silicate hydrate (C-S-H) and calcium hydroxide. These products play an essential role in dimensional stability and interfacial continuity through micro-mechanical interlocking and chemical interaction with dentine [22, 24]. Inadequate moisture may limit hydration and impair setting, while excessive moisture may dilute the material and disrupt its microstructure, explaining the reduced adaptation observed under over-wet conditions [25, 26].

The EDX findings further support the bioactive properties of Bio-C Sealer. The significantly higher calcium and phosphorus levels recorded in this group, indicating an enhanced potential for calcium phosphate deposition at the sealer–dentine interface. This is in agreement with previous studies recording that calcium silicate-based materials release calcium ions that interact with phosphate ions from the surrounding environment, leading to the precipitation of apatite-like structures [26–29]. These findings are in agreement with a previous study demonstrating that bioceramic sealers exhibit favorable biocompatibility and bioactivity, primarily attributed to calcium hydroxide formation and their alkaline pH, which may enhance mineralized tissue formation in spite of the initial inflammatory response [30].

The formation of such mineralized interfacial layers may enhance sealing ability and contribute to long-term interfacial stability. These findings are consistent with other studies recording that the hydration behavior and calcium ion release from calcium silicate-based sealers can be significantly affected by the surrounding chemical environment, which in turn modulates their bioactivity and interfacial performance [31].

The detected higher oxygen signals in Bio-C Sealer are likely associated with the hydration products and ongoing chemical reactions within this material, while the higher carbon levels in AH Plus Jet indicate its organic resin-based composition.

Calcium was also observed in AH Plus Jet, and this may be related to the interaction volume of EDX analysis, which includes the underlying dentine that is naturally rich in calcium. Assuming that the EDX analysis reflects the elemental composition of the interaction volume rather than the sealer alone, these findings should be considered with caution [1, 18].

Variations along the root canal levels were also examined, with obviously greater calcium signals recorded at the apical region in the Bio-C Sealer group. This may be related to the structural variations in dentine, including differences in density and permeability of the tubules, which may affect diffusion of ions and material tissue interaction [30, 32, 33]. The narrower anatomy and reduced diameter of the dentinal tubules at the apical region may enhance localized ion accumulation and promote mineral deposition. In contrast, the lack of obvious regional variations in the AH Plus group is consistent with its non-bioactive nature and limited ionic exchange [29].

The findings of the current investigation are in partial agreement with those recorded by Pelozo et al. [34], who found that dentine moisture conditions affected the adhesive properties of calcium silicate-based root canal sealers. While their study primarily examined the bonding strength and adhesive properties of the interface, the present investigation assessed the interfacial integrity and elemental composition by using (SEM/EDX) analyses and integrated a comparison with an epoxy resin-based (AH Plus Jet) root canal sealer. The enhanced performance of Bio-C Sealer under moist conditions observed in both studies supports the hypothesis that calcium silicate-based root canal sealers perform optimally with residual dentine moisture due to their hydration-dependent setting reactions.

Clinically, these findings highlight the importance of tailoring canal drying protocols in accordance with the type of sealer. Over-dryness may compromise the clinical outcomes of calcium silicate-based sealers, while residual moisture may negatively influence the performance of epoxy resin-based materials. Thus, attaining an optimal residual moisture condition is essential to enhance the clinical performance and treatment outcomes.

### Limitations

1. The in vitro design may not fully replicate the clinical settings, where factors like tissue fluids, microbial activity, and functional loading may affect the sealer performance. Additionally, mechanical properties such as push out bonding strength and micro-leakage were not evaluated. Further studies should integrate clinically representative models and investigate the long-term performance of these materials.
2. The single-cone obturation protocol was applied for all tested groups in order to standardize the experimental conditions. While this approach facilitated a direct comparison between the sealers, it may not reflect the clinical use of AH Plus Jet, which is more commonly applied with compaction-based obturation techniques. Consequently, the thickness and volume of sealer all around the gutta-percha cone may have affected the observed interfacial properties. Additionally, the interfacial integrity was assessed qualitatively by using SEM rather than quantitative assessment of gap formation; thus, conclusions about interface quality should be interpreted with caution. Future studies should include quantitative interfacial analyses, bonding strength evaluation, and long-term investigations under more representative clinical conditions.

## Conclusions

Dentinal moisture conditions significantly affect the interfacial adaptation and elemental characteristics of root canal sealers. AH Plus Jet recorded optimal performance under dry conditions, while the Bio-C Sealer demonstrated improved adaptation and bioactivity under moist conditions. The increased calcium and phosphorus levels recorded in the Bio-C group support its bioactive potential and its ability to promote interfacial mineralization.

## Data Availability

The data supporting the findings of this research are available from the authors upon reasonable request.
